# Simultaneous treatment of trauma patients in a dual room trauma suite with integrated movable sliding gantry CT system: an observational study

**DOI:** 10.1038/s41598-022-20491-2

**Published:** 2022-09-27

**Authors:** Maximilian Kippnich, Maximilian Duempert, Nora Schorscher, Martin C. Jordan, Andreas S. Kunz, Patrick Meybohm, Thomas Wurmb

**Affiliations:** 1 Department of Anesthesiology, Intensive Care, Emergency and Pain Medicine, Subsection Emergency and Disaster Relief Medicine, University Hospital Wuerzburg, Oberduerrbacherstrasse 6, 97080 Wuerzburg, Germany; 2 Department of Orthopaedic Trauma Surgery, University Hospital Wuerzburg, Oberduerrbacherstrasse 6, 97080 Wuerzburg, Germany; 3 Department of Diagnostic and Interventional Radiology, University Hospital Wuerzburg, Oberduerrbacherstrasse 6, 97080 Wuerzburg, Germany; 4 Department of Anesthesiology, Intensive Care, Emergency and Pain Medicine, University Hospital Wuerzburg, Oberduerrbacherstrasse 6, 97080 Wuerzburg, Germany

**Keywords:** Whole body imaging, Trauma

## Abstract

The trauma center of the University Hospital Wuerzburg has developed an advanced trauma pathway based on a dual-room trauma suite with an integrated movable sliding gantry CT-system. This enables simultaneous CT-diagnostics and treatment of two trauma patients. The focus of this study was to investigate the quality of the concept based on defined outcome criteria in this specific setting (time from arrival to initiation of CT scan: tCT; time from arrival to initiation of emergency surgery: tES). We analyzed all trauma patients admitted to the hospital’s trauma suite from 1st May 2019 through 29th April 2020. Two subgroups were defined: trauma patients, who were treated without a second trauma patient present (group 1) and patients, who were treated simultaneously with another trauma patient (group 2). Simultaneous treatment was defined as parallel arrival within a period of 20 min. Of 423 included trauma patients, 46 patients (10.9%) were treated simultaneously. Car accidents were the predominant trauma mechanism in this group (19.6% vs. 47.8%, p < 0.05). Prehospital life-saving procedures were performed with comparable frequency in both groups (intubation 43.5% vs. 39%, p = 0.572); pleural drainage 3.2% vs. 2.2%, p = 0.708; cardiopulmonary resuscitation 5% vs. 2.2%, p = 0.387). At hospital admission, patients in group 2 suffered significantly more pain (E-problem according to Advanced Trauma Life Support principles^©^; 29.2% vs. 45.7%, p < 0.05). There were no significant differences in the clinical treatment (emergency procedures, vasopressor and coagulant therapy, and transfusion of red blood cells). tCT was 6 (4–10) minutes (median and IQR) in group 1 and 8 (5–15.5) minutes in group 2 (p = 0.280). tES was 90 (78–106) minutes in group 1 and 99 (97–108) minutes in group 2 (p = 0.081). The simultaneous treatment of two trauma patients in a dual-room trauma suite with an integrated movable sliding gantry CT-system requires a medical, organizational, and technical concept adapted to this special setting. Despite the oftentimes serious and life-threatening injuries, optimal diagnostic and therapeutic procedures can be guaranteed for two simultaneous trauma patients at an individual medical level in consistent quality.

## Introduction

Managing two patients with multiple injuries arriving at the same time in an Emergency Department is a challenge. In general, patients with multiple injuries benefit from rapid diagnostic work-up and whole-body CT is widely recognized as the first-line diagnostic tool^1,2^. If performed quickly, it may even have a positive effect on the outcome^3–5^. For this purpose, CT scanners are ideally located in or nearby the trauma resuscitation rooms^6,7^.

In many settings, only a single CT-Scanner is available for trauma work-up, necessitating an immediate triage if two patients arrive simultaneously as imaging can only be performed in sequential order. This might cause a significant delay for the individual patient. In order to ensure rapid, almost simultaneous diagnosis of two patients, a two-room trauma suite with an integrated, movable sliding gantry CT-system might be a safe and effective solution. To our knowledge, there are only a few such special constructions worldwide^7–11^. For the concurrent treatment of two trauma patients, two stationary CT examination tables are positioned head-to-head with one sliding gantry CT in the middle capable of moving between rooms. In this setting, two patients can receive a whole-body CT directly one after the other. At the same time, the examination tables can serve as operating tables and each suite as a full-fledged (decentralized) operating room, if necessary and if transportation to the operating theatre is not feasible due the patient’s critical condition.

After 14 years of clinical and scientific experience (2004–2018) with a single-CT trauma resuscitation room using whole-body CT as the first-line diagnostic tool in trauma patients with multiple injuries, we redesigned our trauma suite^12^. Since 2018, our concept is based on a two-room trauma suite with an integrated movable sliding gantry CT-system. A mobile wall separates both rooms to protect the staff and the second patient from radiation.

Directly after introduction of the new concept, we evaluated the process of trauma care and analysed different sub-steps of the overall process^12^. After familiarisation, training and the treatment of more than 500 trauma patients within the first year, we now intend to identify specific performance criteria for patients who were treated simultaneously in the Trauma suite. In order to test the hypothesis that simultaneous admission of patients does not delay trauma management, we evaluated two indicators of treatment quality, i.e. the time intervals from the arrival of a patient in the trauma suite to the initiation of CT-diagnostics and the time interval from arrival to emergency surgery (if needed). Moreover, we analysed invasive procedures and transfusions, resuscitation measures and coagulation therapy in this special setting, as well as patients’ characteristics and their clinical condition at admission.

## Methods

### Protocol design

This study represents a retrospective data analysis based on a chart review. Need for approval was waived by the local ethics committee of the University of Wuerzburg (reference number 2019070102). All methods were performed in accordance with the relevant guidelines and regulations.

We screened all patients with severe trauma admitted to the trauma suite that were treated from 1st May 2019 through 29th April 2020 in the Trauma suite. This corresponds with the annual trauma patient volume of the years 2020 and 2021. Two groups were created: trauma patients, who were treated without a second trauma patient present (group 1) and patients, who were treated simultaneously with another trauma patient (group 2) (both patients in this situation are part of group 2). Simultaneous treatment was defined as parallel arrival within a period of 20 min. Due to the lack of comparable studies, there is no consistent definition.

We collected patient and trauma characteristics (gender, age, American Society of Anesthesiologists-Status (ASA), mechanism of trauma, type of trauma (penetrating/blunt), prehospital Glasgow Coma Scale (GCS) and prehospital treatment, Injury Severity Score (ISS) and the two time intervals, i.e., time to CT (tCT) and time to emergency surgery (tES). Moreover, we evaluated outcome variables (mortality, Revised Injury Severity Classification-II-Score (RISC-II)). These patient characteristics and variables are recorded routinely in the hospital’s clinical information system. From the digital anaesthesia protocol, we recorded the patient status at admission including ABCDE-problems (Table 1), invasive procedures, transfusions, vasopressors and coagulation therapy, if applicable.

**Table 1:** Definitions used to assess the patient’s ABCDE-Status at admission at the trauma resuscitation room.

Airway-problem	Measures to secure the airway or problems with the preclinical established airway device
Breathing-problem	Hypoxia (SpO_2_ < 90%), respiratory rate > 20/min or < 10/min, pneumothorax, problems with the preclinical established pleural drainage
Circulation-problem	Heart rate > 100 bpm, systolic blood pressure < 100 mmHg, heart rate (bpm) > systolic blood pressure (mmHg), critical bleeding, need of catecholamines
Disability-problem	GCS < 15, neurological deficit, seizure, hypoglycemia
Exposure/environment-problem	Hypothermia, pain/need of analgesia, burn

### Setting

**Figure 1:**
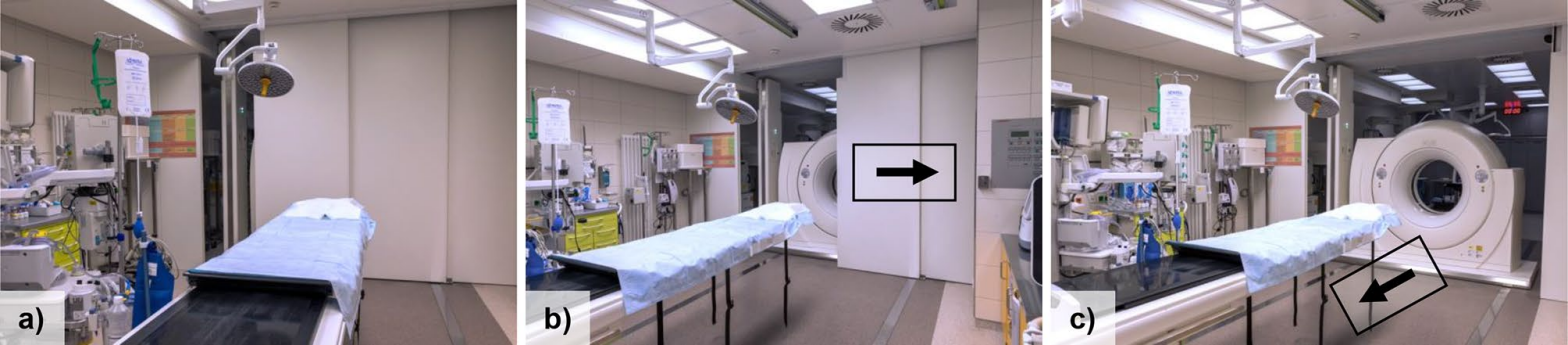
Illustration of the dual-room trauma suite setup with a single sliding gantry-based CT scanner. (**A**) Depicts one trauma suite with removed CT scanner. The sliding door separating both suites while providing radiation protection can be opened (**B**) and the sliding gantry approaches the stationary patient in order to perform the CT scan.

**Figure 2:**
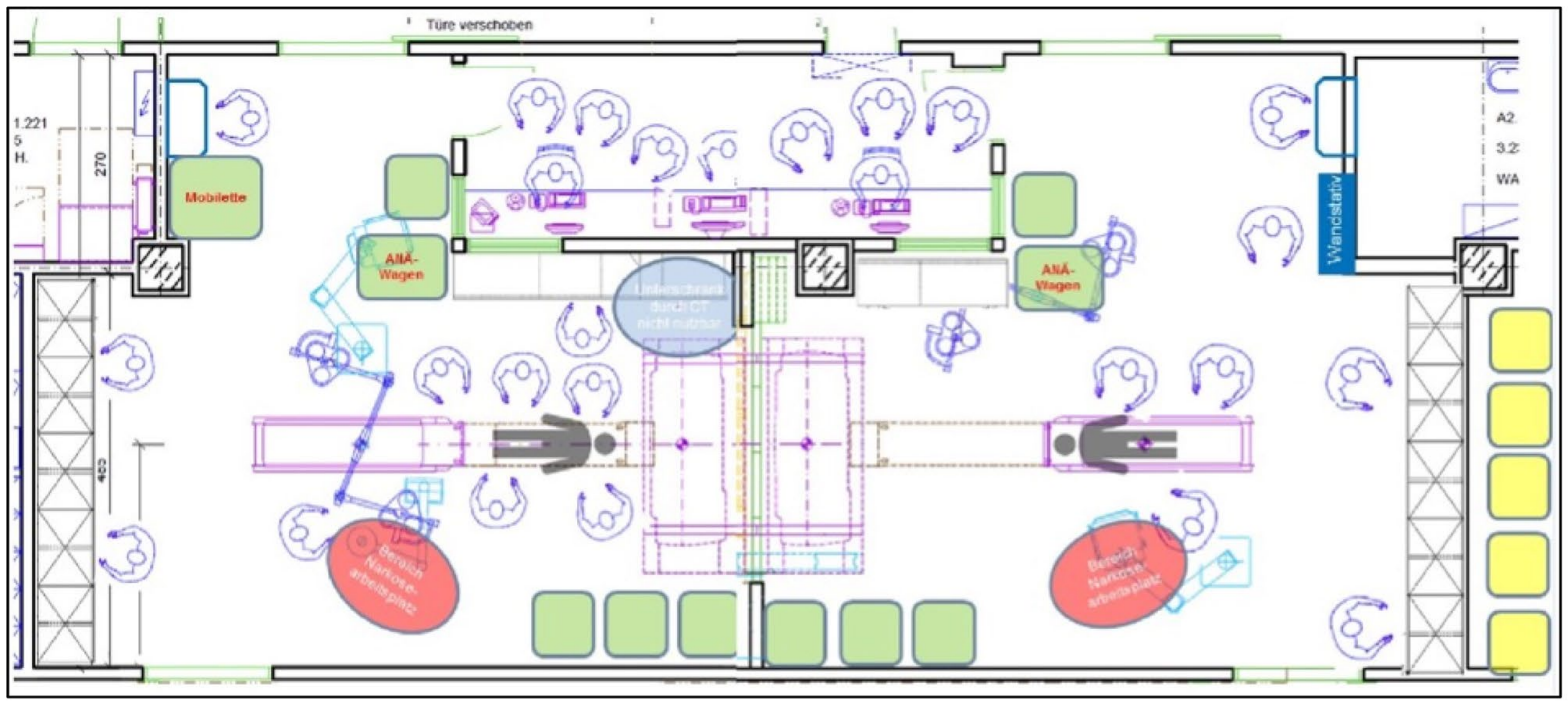
Schematic illustration of the entire trauma suite including the radiology workstation (top view).

The University Hospital Wuerzburg is a certified Level One Trauma Centre. Since 2018, a dual-room sliding gantry CT scanner (SOMATOM Definition Edge, Siemens Healthineers, Erlangen, Germany) (Figs. 1 and 2) is located in a two-room trauma suite for simultaneous treatment of two trauma patients. The installed CT system is a fully certified clinical scanner with applied radiation doses in the range of comparable systems without sliding gantries. With duplicated life support monitors in the control suite and a large scale lead glass window panel providing both radiation protection and visibility, no staff remains in direct proximity to the patient and scanner during image acquisition on a regular basis. If that were necessary, personal protective equipment is available in the suite. The trauma algorithm of our trauma centre, well established since 2004, was adapted to the special requirements of simultaneous treatment of two patients with multiple injuries. Trauma team activation is based on the information provided by the Emergency Medical Service on scene and in particular on the severity of injury (life-threatening) or the mechanism of trauma.

The management of the trauma team represents an interdisciplinary approach with a “leadership group” consisting of a senior anaesthesiologist, senior trauma surgeon, senior general surgeon and senior radiologist^13^. If two patients are scheduled for overlapping or simultaneous arrival, the leadership group coordinates the distribution of personnel and the prioritization of diagnostics and emergency surgery, as well as the additional allocation of resources, if needed. The minimum staffing of the trauma team includes:

- Two anaesthesiologists (senior and resident)
- Two surgeons (senior and resident)
- One trauma surgeon (senior and resident)
- One radiologist (senior)
- Two anaesthesiological nurses, one to two surgical nurses and one radiology technician.

For simultaneous treatment, the trauma team will be augmented accordingly. Specialist consultants (neurosurgery, cardiac and thoracic surgery, urology, paediatric surgery, maxillofacial surgery and ear, nose and throat) are available within 15 min at any time (24/7).

After handover, the patient is transferred from the ambulance stretcher directly onto the examination table of the CT scanner. Before initiating the CT scan, a physical exam is performed according to the priority-based standard of care, and life-threatening conditions are treated immediately according to ATLS^®^ and the department’s Standard Operating Procedures. The leadership group applies the triage rule for whole-body CT to the trauma victim/victims and sets the order of imaging. This triage rule is based on three categories: mechanism of trauma, clinically apparent injuries and vital signs. If one parameter is positive, standardized whole-body CT is initiated^14^.

After the scans have been completed, the algorithm continues with a reassessment followed by further stabilization and planning of transportation to the operating room or to an ICU. Emergency surgery is defined as surgical procedures necessary without any delay to prevent further harm and to improve long-term prognosis. This represents procedures like craniotomy for traumatic brain injury, laparotomy for blunt abdominal trauma, or external fixation of fractures in orthopaedic damage control.

### Methods and measurements

Time to CT (tCT) and time to emergency surgery (tES) were defined as the time interval from the first automatically recorded vital parameter in the Trauma suite (pulse oximetry is applied during the handover) until initiation of the CT scan, respectively of the emergency surgery in the operating theatre. tCT and tES represent the key performance indicators to prove the hypothesis that simultaneous admission does not delay the trauma management of patients with multiple injuries. The calculation of ISS and RISC-II were performed by one trained investigator, in unclear cases a supervisor was put in charge. According to their ISS, patients were divided into three different subgroups (minor injury ISS 0–15, moderate injury 16–24, severe injury 25–75). To assess the patient’s condition on arrival and render this data comparable, the ABCDE-Status was analysed. The definitions used to assess the patient’s ABCDE-Status are shown in Table 1.

### Statistics

There was no normal distribution of data according to the Kolmogorov–Smirnov test. The data are shown as the median with interquartile ranges (IQR, 25–75% percentile). The non-parametric Mann–Whitney rank sum test and Pearson’s chi-squared test was used. P < 0.05 was considered significant. Statistical analyses were performed with IBM SPSS and Microsoft Excel software.

### Ethics approval and consent to participate

Need of ethical approval was waived by the local ethics committee of the University of Wuerzburg (reference number 2019070102).

## Results

In the study period, a total of 521 patients were treated in the trauma suite. Of those, 98 patients were excluded (53 non-trauma patients, 45 transfer from another hospital). Of 423 included trauma patients, 46 patients (10.9%) were treated simultaneously.

### Patient and prehospital trauma characteristics

The prehospital care was performed by an emergency physician and the paramedics of the Emergency Medical Services. Patients’ and prehospital trauma characteristics are shown in Table 2.

**Table 2:** Patients’ and prehospital trauma characteristics (Age = Median (IQR); p = prehospital).

	Single trauma patient treatment *(group 1) (%)*	Simultaneous trauma patient treatment *(group 2) (%)*	p-value
n	377 (89.1)	46 (10.9)	
Male	267 (70.8)	28 (60.9)	0.165
Age	50 (30–71) Median (IQR)	49.5 (28.5–61.5) Median (IQR)	0.157
**ASA status**			
1	208 (55.2)	26 (56.5)	0.862
2	131 (34.7)	15 (32.6)	0.773
3	38 (10.1)	5 (10.9)	0.867
**Mechanism of trauma**			
Car accident	74 (19.6)	22 (47.8)	0.00002
Motorbike	44 (11.7)	4 (8.7)	0.548
Pedestrian	39 (10.3)	1 (2.2)	0.074
Fall > 3 m	70 (18.6)	2 (4.3)	0.015
Others	150 (39.8)	17 (37)	0.711
**Type of trauma**			
Blunt	362 (96.0)	46 (100)	–
Penetrating	15 (4.0)	0 (0)	–
**Prehospital status and treatment**			
(p) Intubation	164 (43.5)	18 (39.0)	0.572
(p) Pleural decompression	12 (3.2)	1 (2.2)	0.708
(p) Cardiopulmonary resuscitation after cardiac arrest	19 (5.0)	1 (2.2)	0.387

### Injury severity scale

Table 3 displays the numerical distribution of the three ISS subgroups.

**Table 3:** Distribution in the three ISS subgroups (0–15 = minor trauma; 16–24 = moderate trauma; 25–75 = severe trauma).

ISS subgroup	Single trauma patient treatment *(n/group 1) (%)*	Simultaneous trauma patient treatment *(n/group 2)* (%)	p-value
0–15	243 (64.5)	35 (76.1)	0.117
16–24	65 (17.2)	7 (15.2)	0.730
25–75	69 (18.3)	4 (8.7)	0.140

### Clinical condition at admission and emergency procedures

Table 4 illustrates the condition of the patients at time of admission to the Trauma suite.

**Table 4:** Patients’ condition at admission to the Trauma suite (for definitions concerning the ABCDE-Status please refer Table 1).

	Single trauma patient treatment *(group 1) (%)*	Simultaneous trauma patient treatment *(group 2) (%)*	p-value
A-Problem	9 (2.4)	1 (2.2)	0.928
B-Problem	28 (7.4)	3 (6.5)	0.824
C-Problem	106 (28.1)	13 (28.3)	0.984
D-Problem	156 (41.4)	15 (32.6)	0.252
E-Problem	110 (29.2)	21 (45.7)	0.023

Emergency procedures, which were performed by the trauma team, are listed in Table 5.

**Table 5:** Emergency procedures performed in the Trauma suite.

	Single trauma patient treatment *(group 1) (%)*	Simultaneous trauma patient treatment *(group 2) (%)*	p-value
Endotracheal Intubation	24 (6.4)	4 (8.7)	0.549
Pleural decompression	20 (5.3)	0 (0)	0.109
Intra-arterial catheter	121 (32.1)	12 (26.1)	0.407
Central venous catheter	68 (18.0)	5 (10.9)	0.225
Vasopressor therapy	98 (26.0)	16 (34.8)	0.205
Coagulant therapy^a^	29 (7.7)	4 (8.7)	0.811
Transfusion of red blood cells	9 (2.4)	2 (4.6)	0.430

^a^Coagulant therapy: calcium, tranexamic acid, coagula factors.

### Time intervals and outcome variables

343 patients (91%) in group 1 and 44 patients (95.7%) in group 2 underwent whole-body CT (p = 0.284). Time to CT was calculated at 6 (4–10) minutes in group 1 and at 8 (5–15.5) minutes in the second group (p = 0.280). In group 1, emergency surgery was performed in 70 patients (18.6%), and in 9 patients (19.6%) in group 2 (p = 0.870). Time to emergency surgery was 90 (78–106) minutes in group 1 and 99 (97–108) minutes in group 2 (p = 0.081).

Table 6 depicts the RISC-II-Score and the observed mortality.

**Table 6:** RISC-II-Score and observed mortality.

	Single trauma patient treatment *(group 1) (%)*	Simultaneous trauma patient treatment *(group 2) (%)*	p-value
RISC-II	0.989 (0.909–0.997)	0.996 (0.987–0.998)	0.001
Death within 24 h	24 (6.4)	2 (4.3)	0.591
Death within 30 days	30 (8.0)	0 (0)	0.047

**Figure 3:**
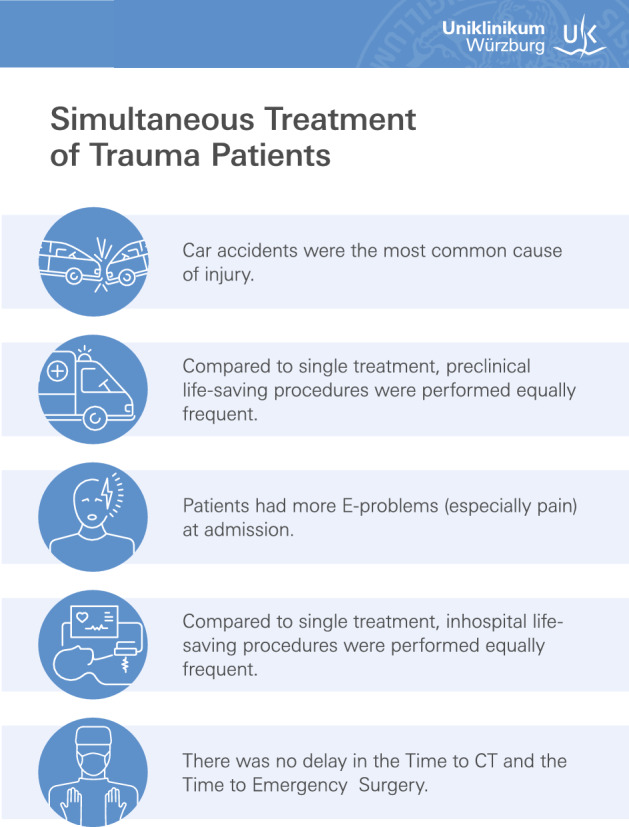
Main outcomes of simultaneous treatment of trauma patients in a dual-room trauma suite with integrated movable sliding gantry CT system compared to single patient treatment.

Figure 3 displays a summary of the main outcomes of simultaneous treatment of trauma patients in a dual-room trauma suite with integrated movable sliding gantry CT system.

## Discussion

The dual-room trauma suite with an integrated movable sliding gantry CT-system represents a significant innovation for the care of patients with multiple injuries. The simultaneous treatment of two trauma patients in this special setting is always a medical, organizational and technical challenge. The goal of a dedicated trauma system is to ensure the highest quality of diagnostics and therapy for patients, even in simultaneous settings. In particular, the immediate application of standardized whole-body CT is a crucial component during the initial assessment of trauma patients.

To better understand the specifics of the simultaneous treatment of two patients in a dual-room trauma suite with integrated movable sliding gantry CT-system, the patients’ characteristics, their medical condition on arrival, invasive procedures and therapies and two significant quality indicators for treatment (tCT and tES) were studied. Of the 423 included trauma patients, 46 (10.6%) were treated simultaneously. A high percentage in both groups received whole-body CT as the first-line diagnostic tool. The number of emergency surgeries were also comparable in both groups. The time to CT and the time to emergency surgery, both being important quality indicators in trauma care, were almost equal in both groups. There were no delayed or missed diagnoses. Importantly, the expected and the observed mortality rate was comparable in group 2 as in group 1. This clearly demonstrates that a well-organized process and a valid structure ensures a consistent quality in the processes of trauma care.

Continuous quality management is also reflected in the improvements of the tCT in our trauma center. In a study performed immediately after the introduction of the new concept (2018), the tCT was found to be about 15 min, compared to a previous study of our trauma center in 2003 where the tCT was about 10 min (single whole-body CT trauma resuscitation room)^1,12^. In this study, the tCT was found to be 6 min (group 1) or 8 min (group 2). In a comparable dual-room setting, Frellesen et al. describes a tCT of 15 min^10^. In the context of acute trauma care, a European multicenter study (REACT-2-study) reported whole-body-CT-imaging to be conducted within 32 min^15^. However, in this study the CT system was not necessarily located within the Trauma suite. The authors acknowledge a potential risk of bias that simultaneously admitted patients might be subjected to. If two patients with multiple injuries are announced, the teams may work under even more pressure in order to have optimal resources for the second (or third etc.) patient available more quickly. Our results are not suggestive for this.

Notably, patients in group 2 suffered more often from car accidents. Car accidents can result in multiple injured patients derived from the same incident. Rarely, such accidents can even lead to a mass casualty incident. These circumstances might explain the high numbers within the group of patients treated simultaneously. A dual-room trauma suite in a Level One Trauma Centre can be a suitable solution for such situations as it offers the capacity to provide high quality trauma care for two severely injured patients at the same time.

The allocation of the patient to the trauma resuscitation room follows the severity of the injuries or the trauma mechanism. This also applies, for example, to patients who do not have serious injuries in the preclinical assessment, but a trauma mechanism that may have caused serious injuries. The rule is then often applied to all potential patients of an accident^16,17^. The relatively high proportion of patients with minor injuries in the simultaneously treated group could be due to such performed assessments.

Prehospital life-saving procedures (endotracheal intubation, pleural drainage, cardiopulmonary resuscitation) were performed equally frequent in both groups. The patients´ condition at admission in the trauma suite assessed by the ABCD-Status was also comparable. Nevertheless, patients in group 2 had significantly more E-problems (especially pain). It might be assumed that prehospital treatment was limited to life-saving procedures and a “load-and-go”-strategy was employed.

In our study, there were no significant differences in the frequency and timing of emergency procedures, central/arterial catheter insertion, use of vasopressors, coagulant therapy and transfusion of red blood cell units performed in the Trauma suite. This demonstrates that these procedures can be applied rapidly and safely, even while treating two patients simultaneously.

The simultaneous treatment of two trauma patients in a dual-room trauma resuscitation suite constitutes a medical, organizational and technical challenge^12^. In this setting, the trauma leadership is a prominent position. For two potentially severely injured patients, staff deployment must be planned, and diagnosis and treatment coordinated and prioritised. Respecting the ATLS^®^-Principles, an interdisciplinary leading team consisting of anaesthesiologists, surgeons, and radiologists is in charge^13^.

We acknowledge that this study has a retrospective design based on a chart review with a limited number of patients. Regardless of the day and time of day, numerous qualified staff are available with little or no delay at our hospital, thus these data may not be applicable to other hospitals.

## Conclusion

To the best of our knowledge, this is the first study examining the specifics of simultaneously treated patients in a dual-room trauma suite with an integrated sliding gantry CT-system.

With a dedicated medical, organizational, and technical concept, the described setting is suitable for treating trauma patients simultaneously ensuring quality standards at the highest possible level.

## Data Availability

The datasets used and analysed during the current study are available from the corresponding author on reasonable request.

## Abbreviations

ASA: American Society of Anesthesiologists-Status
ATLS: Advanced Trauma Life Support
CT: Computed tomography
GCS: Glasgow Coma Scale
ICU: Intensive Care Unit
IQR: Interquartile Range
ISS: Injury Severity Score
RISC-II: Revised Injury Severity Classification-II-Score
SpO2: Peripheral Oxygen Saturation
tCT: Time to CT
tES: Time to Emergency Surgery
